# A Balanced Mixture of Antagonistic Pressures Promotes the Evolution of Parallel Movement

**DOI:** 10.1038/srep39428

**Published:** 2016-12-20

**Authors:** Jure Demšar, Erik Štrumbelj, Iztok Lebar Bajec

**Affiliations:** 1 Faculty of Computer and Information Science, University of Ljubljana, Slovenia.

## Abstract

A common hypothesis about the origins of collective behaviour suggests that animals might live and move in groups to increase their chances of surviving predator attacks. This hypothesis is supported by several studies that use computational models to simulate natural evolution. These studies, however, either tune an ad-hoc model to ‘reproduce’ collective behaviour, or concentrate on a single type of predation pressure, or infer the emergence of collective behaviour from an increase in prey density. In nature, prey are often targeted by multiple predator species simultaneously and this might have played a pivotal role in the evolution of collective behaviour. We expand on previous research by using an evolutionary rule-based system to simulate the evolution of prey behaviour when prey are subject to multiple simultaneous predation pressures. We analyse the evolved behaviour via prey density, polarization, and angular momentum. Our results suggest that a mixture of antagonistic external pressures that simultaneously steer prey towards grouping and dispersing might be required for prey individuals to evolve dynamic parallel movement.

Results from studies of collective behaviour are useful for scientists from many different research fields–from biology, physics and medicine, to computer science^1^,^2^,^3^,^4^,^5^. Because humans behave similarly as groups of animals in a wide repertoire of situations, such as traffic jams and behaviour at large-scale events (e.g. sport games, music concerts), collective behaviour is also interesting from the social studies perspective^3^,^5^.

The literature about collective behaviour contains several hypotheses about why animals, such as schools of fish, flocks of birds, swarms of insects, and herds of ungulates coalesce into groups. Some studies suggest that animal groups may increase the mating and foraging efficiency of their members^6^, or that grouping could save energy because of hydrodynamic or aerodynamic benefits^7^,^8^,^9^.

Probably the most common hypotheses about the evolution of collective behaviour are related to protection from predation^2^,^10^,^11^,^12^,^13^,^14^,^15^. The selfish herd hypothesis suggests that animals form groups in order to reduce their individual domain of danger^16^,^17^,^18^. The confusion effect hypothesis states that a predator attacking a group of visually similar prey might have a hard time tracking and capturing its target^14^,^19^,^20^,^21^,^22^,^23^. The many eyes hypothesis suggests that as the size of the group increases the amount of time an individual has to scan the environment decreases^24^,^25^. And the dilution of risk hypothesis suggests that the chance of a single prey being selected as the predator’s target is lower in larger groups^26^.

Computational models are becoming a frequent tool for studying various hypotheses concerning collective behaviour^2^,^3^,^27^. In computational models genetic algorithms^28^ and genetic programming^29^ are usually used to simulate artificial evolution. Artificial evolution can help us understand which selective pressures may have been the reason for collective behaviour to evolve. Wood and Ackland^30^ used genetic algorithms to tune parameters of the model that was originally presented by Couzin *et al*.^31^ to suggest that predation might promote the evolution of laterally expanded visual perception. Kunz *et al*.^20^ evolved artificial neural networks to show that the presence of a confusable predator might be a sufficient condition for prey individuals to evolve collective behaviour. Using a comparable technique, a similar result was achieved by Olson *et al*.^21^,^22^, who in addition showed that predators may reduce the benefits of prey grouping by attacking peripheral targets and that grouping evolves when the predators attack prey individuals that are located nearby. Morrell *et al*.^17^ showed that complex rules outperform simple ones under a range of predator attack strategies. As a contrast Demšar *et al*.^19^ used genetic algorithms to evolve composite predation tactics and showed that confusion might play an important role in the evolution of these. A recent study by Biswas *et al*.^32^ suggests that the dilution of risk is the most prominent factor for the evolution of clumping and not the confusion effect as suggested by previous research.

In the studies that investigated the evolution of collective behaviour under various predation tactics^17^,^21^,^22^,^32^ researchers were mostly interested in whether prey individuals start to group or not (i.e. whether as a result of the artificial evolution the prey density increased or decreased). As groups of animals in nature move in many different regimes (clumping, swarming, milling, schooling, …)^12^,^33^ and different predation pressures are countered by different responses of prey groups, we can hypothesise that the type of predation tactic has an influence on the type of collective behaviour that evolves.

In this study we focus on how predation from various types of predators influences the evolution of collective behaviour in prey individuals. We let prey individuals evolve their behaviour while experiencing predation from a) predators for which grouping might be a natural response and b) predators for which dispersing might be a natural response. According to previous research there are several predation tactics that pressure prey individuals to evolve grouping behaviour^20^,^21^,^22^,^32^. Two of these are a) attack prey individuals located at the periphery of the prey group (P, periphery) and b) attack the nearest prey individual (N, nearest). Predation tactics that pressure prey towards dispersing (against grouping behaviour), are a) attack the most central prey individual in a prey group (C, centre) and b) high density area attacks (H, density)^21^.

Predators that attack the nearest, the most peripheral or the most central prey individual usually detect, pursue, attack and capture a single prey individual. For example, black seabass, *Centropristis striata*, in attacks on schools of Atlantic silversides, *Menidia menidia*, focus on stragglers when these are available, otherwise they most often target central prey^34^. Predators that focus on nearest prey individuals are typically sit-and-wait, ambush or surprise-attack predators (e.g. largemouth bass, *Micropterus salmoides*^35^ or peregrine falcons, *Falco peregrinus*^36^) who try to minimize energy costs required for prey capture. However, rather than relying on a single predation tactic predators usually adapt their tactic with respect to the prey species. For example, in attacks on either free-swimming whirligig beetles, *Dineutes discolor*, or a constrained group of tadpoles, *Bufo bufo*, largemouth bass, *Micropterous salmoides*, like goldfish, *Carassius auratus*, preferentially attack prey on the periphery^37^. Recent research by Ioannou *et al*.^38^ showed that bluegill sunfish, *Lepomis macrochirus*, when hunting virtual prey disproportionately more often attack prey relatively far from the group centre, but only in the case when prey individuals are moving with relatively low tortuosity. In attacks on groups, on the other hand, prey in groups with a coordinated direction of motion (i.e., with high polarization) were at less risk than their counterparts in unpolarized swarms.

Density attacking predators are usually larger than a single prey individual and do not necessarily detect, pursue, attack and capture a single prey individual, but can attack and capture several prey individuals in a single predation event (e.g. whales). Minke whales, *Balaenoptera acutorostrata*, lunge feed on small schooling fish^39^. Killer whales, *Orcinus orca*, are known to use cooperative hunting^40^,^41^. Basking sharks, *Cetorhinus maximus*, on the other hand, are filter-feeders and feed while cruising at a relatively low and constant speed^42^. In these cases, while the prey may be able to accelerate and manoeuvre at a higher rate than the predator, the difference in size is such that, once the prey is aimed at, its speed is too low to avoid the predator’s large gape^43^. In nature, prey living in groups are often targeted by such predators^41^,^43^,^44^,^45^, and scenarios where prey are subject to multiple predation tactics simultaneously (e.g. attack single peripheral prey individuals, and density attacks) are not uncommon (e.g. multi-species feedings)^46^,^47^,^48^. Exposure to such conflicting, antagonistic predation pressures might have played a pivotal role in the evolution of collective behaviour. For this reason we investigated the type of evolved behaviour when varying exposure to multiple (conforming and antagonistic) simultaneous predation pressures.

## Results

The influence of predation pressures on the evolution of prey behaviour was studied by varying the number of predators using different specific predation tactics. The total number of predators used to obtain results reported in this study was eight, but no significant difference was observed while performing preliminary tests with larger or smaller numbers of predators.

**Figure 1:**
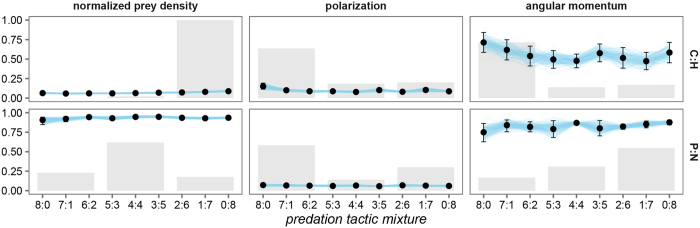
Normalized prey density, polarization, and angular momentum for conforming predation pressure mixtures; P – attack prey individuals located at the periphery of prey groups, N – attack the nearest prey individual, C – attack the most central prey individual in a prey group, and H – high density area attacks. The predation pressure mixture ratio *a:b* denotes the number of predators using a specific predation tactic, e.g. in the case of C:H, 0:8 (top row, right side of the plot) all predators (eight) use high density area attacks. Points and whiskers represent the estimated posterior means and 95% posterior confidence intervals. Individual draws from the posterior distributions are connected with lines to visualize posterior uncertainty and aid in the interpretation of how the means vary across the predation pressure mixtures. To summarize the results, the predation mixtures were grouped into groups of three: predation pressure predominantly from centre (C:H) or periphery (P:N) attacking predators (8:0, 1:7, 2:6), balanced pressure (5:3, 4:4, 3:5), and predation pressure predominantly from high density area (C:H) or nearest prey individual (P:N) attacking predators (6:2, 7:1, 0:8). The shaded bars show, for each group, the probability that that group has the highest mean. These probabilities were estimated with draws from the posterior distributions in which each group member had an equal probability of being selected. That is, each predation mixture was weighted equally.

According to previous studies^20^,^21^,^22^,^32^ predation pressure from predators that attack the most central prey individual in a prey group (C) or use high density area attacks (H) should promote the evolution of the tendency to disperse in prey individuals. On the other hand, predation pressure from predators that attack prey individuals located at the periphery of the prey groups (P) or the nearest prey individual (N) should lead to the evolution of the tendency to group. As from the point of view of the expected outcome in both cases the two predation tactics agree, regardless of the number of predators using a specific tactic (dispersion for combination C:H, grouping for P:N), we call such predation pressures conforming. Our results (Fig. 1) support previous studies. With conforming pressures towards dispersing by centre and density attacking predators the mean normalized prey density consistently stayed below 0.125, regardless of the number of predators using a specific tactic. On the other hand, with conforming pressures towards grouping by periphery and nearest prey individual attacking predators the mean normalized prey density remained above 0.75, regardless of the number of predators using a specific tactic.

Couzin *et al*.^31^ introduced the measures of polarization and angular momentum. They express the degree of consensus in a common heading of the group (polarization), and the degree of rotation of the group about the group’s centre (angular momentum). By means of these two measures Couzin *et al*.^31^ defined four collective dynamical behaviours: swarm (low polarization and low angular momentum), torus or milling (low polarization and high angular momentum), dynamic parallel group (high polarization and low angular momentum), and highly parallel group (very high polarization and low angular momentum)^31^.

Conforming predation pressures (combinations C:H and P:N in Fig. 1) resulted in behaviours with medium to high angular momentum and low polarization. While polarization was low for all pressure mixtures, its mean was highest when predation pressure came predominantly from centre or periphery attacking predators. Predation pressures for which the response was grouping led to consistently high angular momentum with low variability. Here attacks directed predominantly on the nearest prey individual gave rise to the highest mean angular momentum. The high angular momentum combined with the consistently low polarization suggests the evolution of milling behaviours. Pressure towards dispersing, on the other hand, resulted in substantially lower angular momentum with a higher variability. Here, domination by centre attacking predators induced the highest mean angular momentum. As polarization was low the medium to high angular momentum with high variability suggests the evolution of either swarming or milling behaviours. Note here that even in cases where polarization was the highest it was not high enough to suggest the observed behaviour could be classified as polarized.

**Figure 2:**
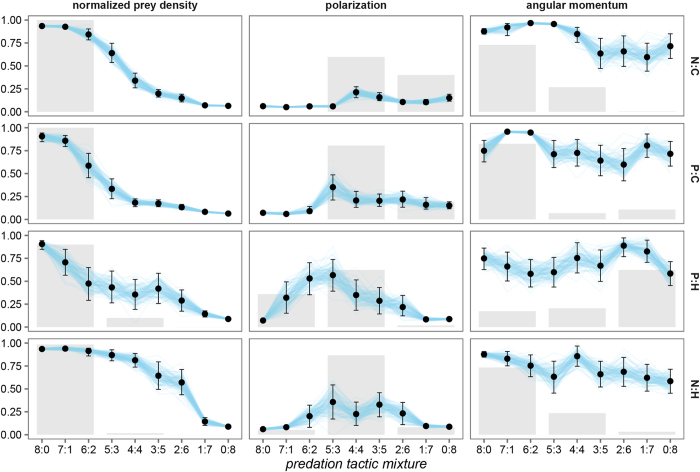
Normalized prey density, polarization, and angular momentum for antagonistic predation pressure mixtures; P – attack prey individuals located at the periphery of prey groups, N – attack the nearest prey individual, C – attack the most central prey individual in a prey group, and H – high density area attacks. The predation pressure mixture ratio *a:b* denotes the proportion of predators using a specific predation tactic, e.g. in the case of N:C, 0:8 (top row, right side of the plot) all predators (eight) attack the most central prey individual in a prey group. Points and whiskers represent the estimated posterior means and 95% posterior confidence intervals. Individual draws from the posterior distributions are connected with lines to visualize posterior uncertainty and aid in the interpretation of how the means vary across the predation pressure mixtures. To summarize the results, the predation mixtures were grouped into groups of three: low degree of antagonism in predation pressures with predominant pressure from predators that force prey into grouping (8:0, 1:7, 2:6), high degree of antagonism in predation pressures (5:3, 4:4, 3:5), and low degree of antagonism in predation pressures with predominant pressure from predators that force prey into dispersion (6:2, 7:1, 0:8). The shaded bars show, for each group, the probability that that group has the highest mean. These probabilities were estimated with draws from the posterior distributions in which each group member had an equal probability of being selected. That is, each predation mixture was weighted equally.

In the case of antagonistic predation pressures (combinations N:C, N:H, P:C and P:H, see Fig. 2) the highest mean normalized prey density emerged when predation pressure came predominantly from the nearest or the most peripheral prey individual attacking predators (predators that in a conforming setting pressure prey into grouping; mixtures 8:0, 7:1, 6:2). On the other hand, domination by predators for which the expected outcome is dispersing (mixtures 2:6, 1:7, 0:8) led to the lowest mean normalized prey density. Domination by either pressure to group or pressure to disperse can be interpreted as a low degree of antagonism in predation pressures. A high degree of antagonism, where neither the pressure to disperse nor the pressure to group dominates (mixtures 5:3, 4:4, 3:5), led to the evolution of low to high mean normalized prey density. The lowest in the case of periphery and centre attacking predators (P:C) and highest in the case of nearest prey individual and high density area directed attacks (P:H).

Evolution under antagonistic predation pressures resulted in medium to high angular momentum and low to medium polarization. Compared to the case of conforming predation pressures, there was a considerably higher variability in the values of angular momentum and polarization, suggesting a wider range of evolved behaviours. In all but the P:H case, predation pressure predominantly from predators that result in prey individuals that favour grouping gave rise to the highest angular momentum. In the P:H case, on the other hand, it was highest when the pressure to disperse dominated. Note that angular momentum was never the highest when antagonism in predation pressures was high (predation pressure mixtures 5:3, 4:4, 3:5). High antagonism, however, always generated the highest mean polarization.

Observing all three parameters in unison (see Supplementary information, Figure S4) suggests that increasing the pressure towards dispersing from centre or high density area attacking predators leads to a general decrease in normalized prey density, angular momentum and polarization (top and right portion of the figure). An increase in pressure towards grouping from predators that attack the nearest or the most peripheral prey individual, on the other hand, causes an increase in density and a favouring of higher momentum with low polarization (bottom and left portion of the figure). Higher values of polarization with low momentum and medium density emerge when the mixture in pressures towards and against grouping (towards dispersing) is somewhat balanced. This suggests a possible emergence of polarized behaviour.

**Figure 3:**
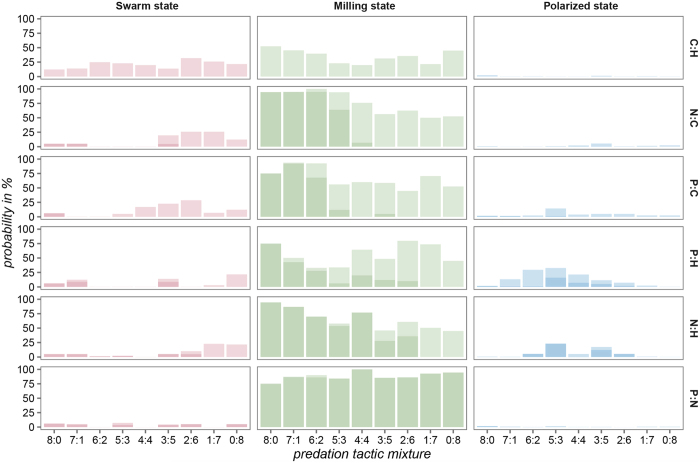
The probability of observing a specific collective state at the end of the simulation run for all predation pressure mixtures; P – attack prey individuals located at the periphery of prey groups, N – attack the nearest prey individual, C – attack the most central prey individual in a prey group, and H – high density area attacks. The shading denotes cases with low (light shading) and high normalized prey density (dark shading). The predation pressure mixture ratio 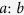 denotes the proportion of predators using a specific predation tactic, e.g. in the case of C:H, 8:0 (top row, left side of the plot) all predators (eight) attack the most central prey individual in a prey group.

Note that polarization and angular momentum reported in Figs 1, 2 and S4, although indicative of the resulting behaviour, cannot be used for such a classification directly, because they were computed as weighted sums of polarization and angular momentum of individual groups and averaged over a number of frames (see Methods). Therefore, to further analyse the influence of predation pressures on the evolution of prey behaviour, we categorized the behaviour of individual groups with respect to their polarization and angular momentum in the last frame of of every simulation run. Here we followed Tunstrøm *et al*.^49^, who used polarization and angular momentum and a simple threshold to categorize the behaviour of groups of golden shiners, *Notemigonus crysoleucas*, into one of three collective states; swarm, milling, and polarized state. Based on that we computed the probability of observing a particular collective state (see Methods). This analysis provides further insight into what kind of behaviour evolves under a particular predation pressure mixture ratio. Figure 3 confirms what was suggested by data presented in Figs 1, 2 and S4: the probability of observing the swarm state is present mainly in cases when prey individuals evolved while under predation pressure from centre or high density area attacking predators. The probability of observing a swarm state is the highest when prey individuals evolved under conforming pressures towards dispersing by centre and density attacking predators. Overall, the probability of observing a milling state dominates, and is high in most cases, except when: a) prey individuals evolved under conforming pressures towards dispersing by centre and density attacking predators, and b) prey individuals evolved under certain antagonistic pressures. On the other hand, evolution under antagonistic pressures produced the highest probability of observing a polarized state. This suggests that a mixture of antagonistic pressures that simultaneously steer prey towards grouping and dispersing might be required for prey individuals to evolve parallel movement.

Visual inspections of the evolved behaviours confirmed that the domination of predation pressures towards dispersing led prey to evolve behaviours where they in general tend to disperse. This tendency to disperse at times leads to the wall-following of the living area border and thus while keeping a low density causes a relatively high angular momentum (see Supplementary information, Video S1). As this is classified as milling, the probability of observing the milling state dominates in Fig. 3. The solution being classified as milling is probably irrelevant for biological organisms as it is determined by the artificial ecology used for this computational study (i.e. crossing the living area border would sooner or later be lethal for the prey individual). Nevertheless, in an experiment with zebrafish, *Danio rerio*, the distribution of the positions detected in the tank showed that the fish avoid the centre of the tank and the higher probability of presence is along the walls^50^, which might be indicative of a similar form of wall-following. On other occasions with low density the evolved behaviour resembled collective motion typically identified as swarming, associated with low angular momentum, and low polarization (see Supplementary information, Video S2). Predation pressure predominantly from predators that push prey into grouping led prey individuals to evolve behaviours that usually resulted in high density, high angular momentum and low polarization, values typically associated with milling (see Supplementary information, Video S3). On few occasions the evolved behaviour resulted in high density, low angular momentum and low polarization, indicative of high density swarming (see Supplementary information, Video S4). As suggested by data presented in Figs 2 and 3 behaviours with higher values of polarization emerged mostly when there was a high degree of antagonism in predation pressures (i.e. when there was a balanced mixture of pressure towards and against grouping). Here prey individuals often evolved behaviours that resulted in parameter values typical for dynamic and highly parallel motion; medium to high normalized density, low to medium angular momentum and medium to high polarization (see Supplementary information, Videos S5–S7).

## Discussion

Previous research that used evolutionary computational models to study the evolution of collective behaviour^17^,^20^,^21^,^22^,^32^ suggests that prey grouping might have evolved as a defensive mechanism against predation. Most of the existing studies were principally interested in whether prey evolve a) grouping behaviour (defined as an increase in prey density) or b) dispersing behaviour (defined as a decrease in prey density). Our study corroborates previous findings in that prey density is high when prey individuals evolve while under predation pressure from predators for which grouping might be a natural response (attack peripheral prey individuals, or attack the nearest prey individual), and low when prey individuals evolve while under predation pressure from predators for which dispersing might be a natural response (attack the most central prey individual, or attack high density areas).

Groups of animals in nature, however, move in many different fashions (clumping, swarming, milling, schooling, …)^12^,^33^ and different predation pressures are countered by different responses. As these responses are experience dependant^51^,^52^, we can hypothesise that if collective behaviour evolved as an anti-predator response it might as well have been shaped by the predation pressures the prey faced. In this study we therefore expanded on previous research by focusing on how predation from various types of predators might influence the evolution of collective behaviour in prey individuals. More specifically we investigated the influence of antagonism between predation pressures towards and against grouping (towards dispersing) on the type of evolved collective behaviour (evaluated via prey density, polarization and angular momentum^31^). Our results suggest that when prey individuals evolve while under conforming predation pressures (either towards grouping or towards dispersing) the resulting behaviour has low polarization and medium to high angular momentum. When prey individuals evolve while subject to antagonistic predation pressures (towards grouping and towards dispersing, simultaneously) density and angular momentum increase with the number of predators forcing prey into grouping and decrease with the number of predators that force prey individuals into dispersing. Polarization, on the other hand, is highest when antagonism in predation pressures is high.

Our results therefore suggest that antagonism might have played an important role in the evolution of collective behaviour; that antagonism from predation pressures, environmental or internal factors could have been responsible for the evolution of a multitude of different behaviours. They could also indicate that in nature the evolution of highly polarized movement might be a result of the co-evolution of prey evasion and composite predator attack tactics^19^. Another possibility is that not only variation in swimming performance^53^, but also the amount of variation in group behaviours might be linked to environmental factors. This supports the hypothesis that ecological constraints may shape the process used to regulate activity in many biological species^54^. Indeed, evolution of group responses to predation in nature is not universal, and different species might evolve very different responses to predation. This is not restricted to fish schools, as it is evident also in avian group behaviour, where the magnitude in variation of behaviours has always been a major puzzle. Why do so few bird species that fly together display organized behaviour, and why do even closely related species display major differences in flocking behaviour^2^. For example, pigeons are more closely related to swifts than they are to starlings^55^, but they flock much more like starlings. Similarly, geese are more closely related to chickens than they are to cormorants^55^, but they fly like cormorants [F.H. Heppner, *personal communication*, February, 2016]. In view of our results one possible explanation for these observations might be that organized flight has evolved independently, and several times, and the differences in behaviour might have emerged because, although closely related, individual species were subject to different pressures.

In marine ecology Rieucau *et al*.’s recent studies suggest that collective anti-predator responses in herring increase with the density of the school^56^, and the number of sensory cues^57^,^58^. Our results suggest that the increase might be accentuated by the conflict in sensory cues. This corroborates recent results by Lemasson *et al*.^59^, who suggest that the benefits of coordinated motion are context dependent, i.e. they can potentially reduce the time prey individuals spend in dangerous areas and help them to avoid becoming isolated, yet such movement patterns can also alleviate predator confusion during a directed attack. It is important to note that exposure to predators affects prey both directly and indirectly and that plasticity in response to risk might relate to an individual’s willingness to take risks^60^. As our model is heterogeneous in the behaviour of prey individuals, our findings seem to suggest that this individuality in susceptibility to predation risk, might inevitably also lead to changes in the behaviour of the group.

In summary, while the dilution of risk might be sufficient for prey individuals to evolve grouping^32^, and predator confusion might lead prey individuals to evolve swarming^20^,^21^,^22^, our results suggest that exposure to antagonistic predation pressures might be a necessary requirement for prey individuals to evolve parallel movement. This could indicate that the direction of evolution (grouping or dispersing) is not A versus B, but a labile result—whether grouping or dispersing evolves depends on a) the nature of the group, and b) the pressures that the group finds itself facing.

## Methods

Our individual based model consists of two types of artificial animals—predators and prey. They coexist in a two dimensional environment confined by a circular living area, with their positions and headings at time instant 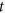 given by 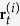, 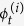. Following previous research^61^,^62^,^63^,^64^,^65^, the behaviour of every artificial animal in our model is governed by fuzzy logic^66^ via a fuzzy-rule-based system^67^. A fuzzy-rule-based system enables the use of linguistic if-then rules to describe the behaviour of the artificial animals. It is specified via a fuzzy knowledge base, which consists of a fuzzy database and a fuzzy rule base. The database lists all input and output variables, as well as the linguistic terms (e.g. near, far, …) that can appear in if-then rules. In addition it includes information necessary for fuzzy reasoning, i.e. the method for transforming crisp data into fuzzy sets (fuzzification), the interpretation of logical connectives necessary for fuzzy reasoning, and the method for converting the fuzzy result into a real action (defuzzification). The fuzzy rule base on the other hand comprises the list of if-then rules that are assumed to be joined by the connective ‘also’, so multiple rules can fire simultaneously. When used in combination with artificial evolution a fuzzy-rule-based system is called a genetic fuzzy system^68^,^69^,^70^.

The fuzzy knowledge base of the predators was set following previous research^64^ (see Supplementary information for details). In the case of prey individuals we, for reasons of simplicity, locked the fuzzy database and evolved only the fuzzy rule base (see Supplementary information for details), but given that fuzzy-rule-based systems are deemed universal approximators^71^ this still provides the opportunity to potentially discover a wide repertoire of behaviours.

**Figure 4:**
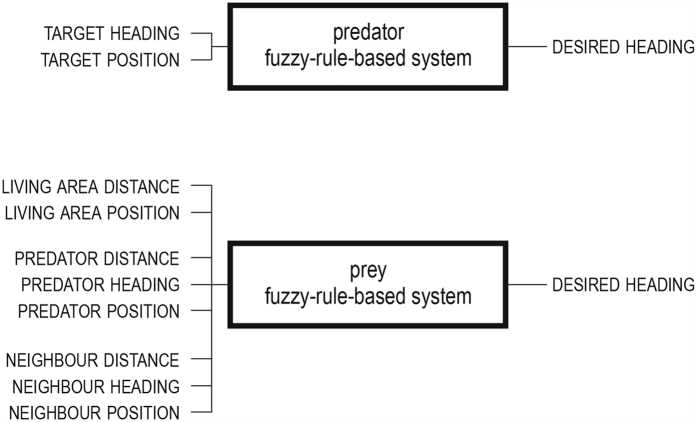
A schematic showing the predator and prey fuzzy-rule-based system’s input and output variables.

At every update step the fuzzy-rule-based systems (i.e. the pre-set fuzzy knowledge base in the case of predators, and the evolving fuzzy knowledge bases in the case of prey individuals) were used to compute the new heading (see Fig. 4) and position of every artificial animal as:


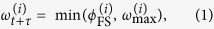



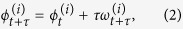



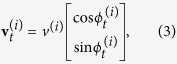



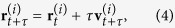


where 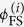 is the desired heading in the local coordinate frame of individual 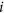 returned by the corresponding fuzzy-rule-based system, 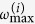 is its manoeuvrability and 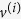 is the animal’s speed. See Supplementary information, Table S3 for a full list of parameters. The following sections provide more details about the evolutionary process and analysis of the evolved behaviour. For more details about the implementation of the predator and prey artificial animal see Supplementary information.

### Evolutionary process

Most of the previous studies concerning the evolution of collective behaviour^20^,^21^,^22^,^30^ used a genetic algorithm, with clearly defined generational boundaries. This is the most frequently used application of genetic algorithms, where in every generation the whole population of potential solutions to the problem is evaluated via simulation in order to evaluate the fitness (assess the quality) of every individual solution. The fitness is then used for selection, followed by reproduction and mutation so that the whole population of possible solutions is created anew and is defined as a new generation. Additionally, with the only exception of Wood & Ackland^30^ in most of the previous studies^20^,^21^,^22^ all of the prey individuals behaved in exactly the same way—the prey groups were homogeneous.

Some recent studies suggest that heterogeneous groups might evolve a different behaviour in an algorithm mimicking artificial evolution^72^. Others suggest that heterogeneous groups might be necessary to achieve a more ‘natural’ behaviour^73^, and that differences among individuals might be essential for group coordination^74^,^75^. For this reason in our approach, similar to Biswass *et al*.^32^, selection, followed by reproduction and mutation are part of the simulation so that there is no clear generational boundary. When a prey individual (a potential solution to the problem) in our system dies, two of the remaining prey individuals are selected based on their current fitness, and via reproduction and mutation a new prey individual is created (a new potential solution to the problem). Since every prey individual is governed by its own fuzzy-rule-based system, this essentially makes the prey group heterogeneous.

The fitness of a prey individual was evaluated via its energy level 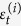, which encodes the individual’s capability to stay in the designated living area, avoid collisions with other prey individuals, and successfully avoid predation. When a new prey individual was created, it was assigned an initial level of energy, 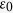, and with every update step the energy level was increased by 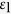. As in the case of Kunz *et al*.^20^ inter-prey collisions were penalized to promote collision avoidance, i.e. in the event of a collision the energy level of the involved prey individuals was decreased by 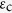. Similarly, to promote staying inside the living area, wandering outside of it (i.e. 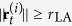) was penalized by 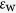. A prey individual died if its energy level depleted to 0 or was marked as captured by a predator (see Supplementary information). Since a new prey individual was created (i.e. it appeared at a random location heading in a random direction) whenever one died, the number of prey individuals was constant throughout the entire evolution.

Individual predators appeared at random time instants at random locations outside of the living area. Their initial heading was towards the centre of the living area, so as to promote the speed of convergence of the evolutionary algorithm^22^. High density area attacking predators hunted until their hunt duration elapsed. Single-target (neatest, centre, and periphery) attacking predators hunted until they caught the currently targeted prey individual, or until their hunt duration elapsed. Once a predator finished its hunt, it was removed, and re-appeared after a random time interval. The initial delay before a predator first appeared, and the time interval before it re-appeared after a hunt were uniformly distributed on the predator re-appearance time interval, 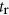. At maximum eight predators were simultaneously present at one time instant.

In order to investigate the type of evolved behaviour when varying exposure to conforming and antagonistic predation pressures we ran individual evolutions while varying the number of predators using a specific predation tactic. In total we ran 54 experiments with different conditions (mixtures of different predation pressure combinations). Each evolutionary run lasted 10.000.000 update steps and was repeated 20 times.

### Analysis of the evolved behaviour

**Figure 5:**
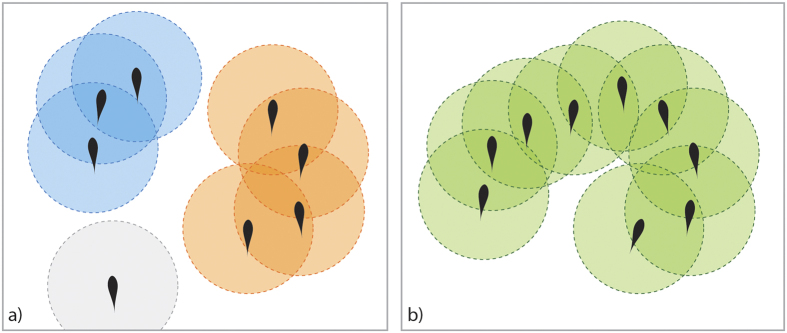
Prey individuals split into groups based on direct and indirect influence; (**a**) one straggler and two proper groups; the straggler does not see any prey individual from either of the two groups, and no member from one group is able to see neither the straggler nor any prey individual from the other group; potential influence (direct or indirect) is limited to members of the same group only; (**b**) one proper group where all members can potentially (directly or indirectly) influence each other’s behaviour.

To classify the evolved behaviour we ran five separate simulation runs where on each occasion the artificial world comprised only the prey individuals from the last update step of the corresponding evolutionary run. This was done to preclude the possibility of the behaviour being classified as collective, when in reality all prey are individually trying to escape the predator in a common direction, hence we analysed the evolved behaviour with no predator present. Each simulation run lasted 1800 update steps and on each occasion the type of behaviour was analysed after it reached a dynamically stable state (i.e. after 900 update steps). For the analysis we turned to the observation of density^22^, polarization, and angular momentum^31^, properties that allow for the categorization of the type of collective behaviour^22^,^27^,^30^,^31^,^49^. Density, eq. (5), can be used to assess the degree of grouping, or clumping. Polarization, eq. (8), express the degree of consensus in a common heading. Angular momentum, eq. (9), the degree of rotation of the group about the group’s centre. Together they can be used to assess the type of collective behaviour (i.e. swarm, torus, dynamic parallel group, or highly parallel group)^31^. The quantities were recorded over the remaining 900 update steps of a simulation, and their individual averages were used as an indication of the evolved behaviour. Normalized prey density


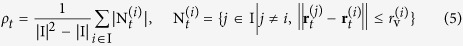


where 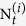 is the neighbourhood of prey individual 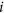, was recorded on a global level. However, in view of recent research^76^, which emphasizes the importance of calculating the observed quantities on a group-by-group basis, for polarization and angular momentum the prey individuals were first split into groups based on direct and indirect influence (see Fig. 5)^77^, where


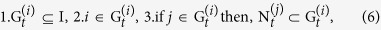


is a recursive definition of a group of prey individuals. Prey individuals pertaining to groups of size one, 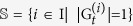, are termed stragglers^77^ and proper groups can be defined as


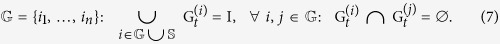


Polarization and angular momentum were computed based on proper groups only and in order to diminish the possible bias induced by many small groups the two quantities at time instant 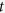 were computed as weighted averages, where group size was used as the weighting function. Hence polarization was computed as


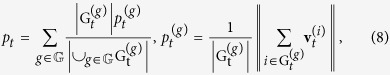


and angular momentum as


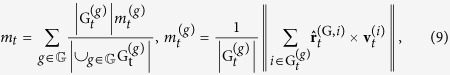


where 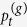 and 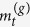 are the polarization and angular momentum of group 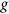, respectively and


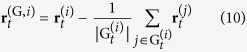


is the relative position of prey individual 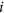 with respect to the centroid of its group.

### Statistical analysis

The main goal of our experiments (evolution + simulation) was to investigate how the mean of three metrics of interest (normalized prey density, polarization and angular momentum) varies across six different pairs of the four predation tactics (C:H, N:C, P:C, P:H, N:H, P:N) and nine different predation mixtures (8:0, 7:1, …, 0:8).

Each combination of the three dimensions above is considered a separate experiment. The experiments are not deterministic—both the evolution and simulation are stochastic in nature and therefore a source of (random) measurement error. To estimate and account for this, each experiment consisted of *n* = 20 different evolutionary runs (iterations), each followed by n = 5 simulation runs (repetitions). The metrics also vary across individual frames, however, low variability and a relatively high number of frames (900) leads to a negligible standard error, therefore, instead of modelling individual frames, the average across all frames was used.

For each experiment separately, we used the following Bayesian hierarchical model to estimate the mean:


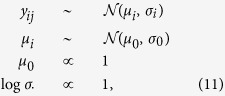


where 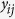 is the metric measurement for the 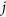-th repetition of the 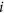-th iteration. That is, we model each iteration with its own distribution with potentially different means 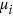 and standard deviations 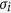. These means are assumed to be drawn from a population of means, with grand mean 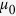, which is what we are interested in estimating. Flat (improper) priors are placed on the (hyper-)parameters. Note that we are only interested in the mean, so the normal model is adequate, although the data are not normally distributed.

We used the Stan tool for Bayesian inference to draw samples from the posterior distribution^78^. Each model was run for 500 warm-up and 5000 sampling iterations, which was sufficient to reduce approximation errors to negligible levels.

In addition to analysing how a specific predation mixture influences the mean of the three metrics (normalized prey density, polarization, angular momentum) we also categorized the behaviour in the last frame (1800) of each simulation run. The categorization was executed on a group-by-group basis following Tunstrøm *et al*.^49^, who defined that a group is in: the polarized state (O) when the group’s polarization > 0.65 and angular momentum < 0.35; the milling state (M) when polarization < 0.35 and angular momentum > 0.65; and the swarm state (S) when polarization < 0.35 and angular momentum < 0.35. Outside these ranges it is said to be in transition (T). In addition, a threshold was used to sub-categorize collective states O, M and S as either low < 0.5 or high density > 0.5. The probability of observing a specific collective state was computed by counting the number of groups in that specific collective state over all evolution iterations and simulation run repetitions of an experiment. To diminish the possible bias induced by small groups, each group contributed only a share proportionate to the size of the group. For example, if 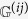 denotes the set of proper groups at the end of iteration 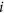, repetition 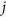 and 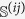 the corresponding subset of proper groups that are in the swarm state, the probability of observing the swarm state was computed as


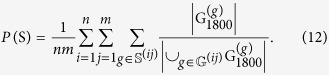


## Additional Information

**How to cite this article**: Demšar, J. *et al*. A Balanced Mixture of Antagonistic Pressures Promotes the Evolution of Parallel Movement. *Sci. Rep.* **6**, 39428; doi: 10.1038/srep39428 (2016).

**Publisher's note:** Springer Nature remains neutral with regard to jurisdictional claims in published maps and institutional affiliations.

## Supplementary Material

Supplementary Video S1

Supplementary Video S2

Supplementary Video S3

Supplementary Video S4

Supplementary Video S5

Supplementary Video S6

Supplementary Video S7

Supplementary Information
