# Supplementary material for: A Balanced Mixture of Antagonistic Pressures Promotes the Evolution of Parallel Movement
Source: Sci Rep. 2016 Dec 20;6:39428. doi: 10.1038/srep39428 (PMC5171647; doi:10.1038/srep39428)
Supplement: Supplementary Information [file srep39428-s8.pdf]

SUPPLEMENTARY INFORMATION FOR
A Balanced Mixture of Antagonistic Pressures Promotes the Evolution of Parallel Movement

Jure Demšar[1,*], **Erik Štrumbelj**[1], **and Iztok Lebar Bajec**[1]

[1]Faculty of Computer and Information Science, University of Ljubljana, Slovenia
[*]jure.demsar@fri.uni-lj.si

ABSTRACT

A common hypothesis about the origins of collective behaviour suggests that animals might live and move in groups to increase their chances of surviving predator attacks. This hypothesis is supported by several studies that use computational models to simulate natural evolution. These studies, however, either tune an ad-hoc model to 'reproduce' collective behaviour, or concentrate on a single type of predation pressure, or infer the emergence of collective behaviour from an increase in prey density. In nature, prey are often targeted by multiple predator species simultaneously and this might have played a pivotal role in the evolution of collective behaviour. We expand on previous research by using an evolutionary rule-based system to simulate the evolution of prey behaviour when prey are subject to multiple simultaneous predation pressures. We analyse the evolved behaviour via prey density, polarization, and angular momentum. Our results suggest that a mixture of antagonistic external pressures that simultaneously steer prey towards grouping and dispersing might be required for prey individuals to evolve dynamic parallel movement.

Individual based model

The behaviour of the artificial animals—predators and prey—in our individual based model is governed by fuzzy logic[1] via fuzzy-rule-based systems[2]. Every fuzzy-rule-based system is specified via a fuzzy knowledge base, which is made from two parts—a data base and a rule base. The rule base lists if-then rules that describe the behaviour of the artificial animal in question. The rules are assumed to be joined by the connective 'also', so multiple rules can fire simultaneously. The antecedent and consequent parts of individual if-then rules use linguistic terms (near, far, left, right, ...), which are defined in the data base of the fuzzy-rule-based system. In addition the data base includes information necessary for fuzzy reasoning (see Table S1), i.e. the method for transforming crisp data into fuzzy sets (fuzzification), the interpretation of logical connectives necessary for fuzzy reasoning, and the method for converting the fuzzy result into a real action (defuzzification). For a detailed description of how a fuzzy rule base is evaluated the reader is invited to refer to[3–5].

In our model, at every update step the fuzzy-rule-based systems (i.e. the pre-set fuzzy knowledge base in the case of predators, and the evolving fuzzy knowledge bases in the case of prey individuals) were used to compute the new heading and position of every artificial animal. Table S3 shows the values of all our model's parameters, while the following sections provide more details about the implementation of the predator and prey artificial animal.

Predators

The predators in our model were hand-tuned and their behaviour was not subject to evolution. Their fuzzy knowledge base (see Figure S1) was set as in previous research[6], with the only difference being that the predators moved at a constant speed.

Table S1. Fuzzy data base settings common to predator and prey fuzzy-rule-based systems.

Description	Default value
Fuzzification	Singleton
Fuzzy conjunction	Product
Fuzzy disjunction	Probabilistic sum
Fuzzy implication	Product
Fuzzy aggregation	Probabilistic sum
Defuzzification	Center of gravity

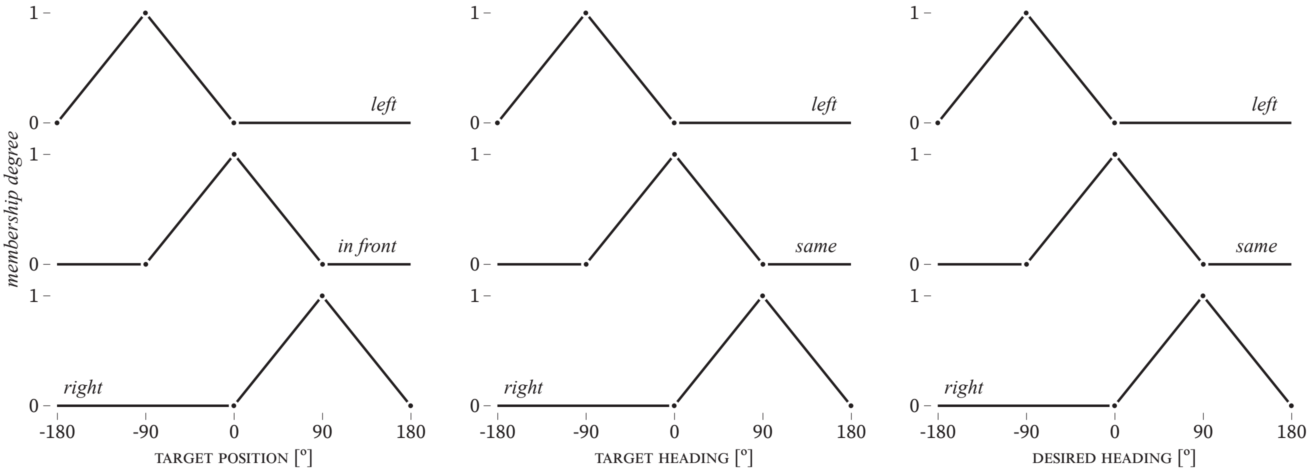

R1: **if** (TARGET POSITION **is** *right*) **then** (DESIRED HEADING **is** *right*)
R2: **if** (TARGET POSITION **is** *left*) **then** (DESIRED HEADING **is** *left*)
R3: **if** (TARGET POSITION **is** *in front*) **and** (TARGET HEADING is *right*) **then** (DESIRED HEADING **is** *right*)
R4: **if** (TARGET POSITION **is** *in front*) **and** (TARGET HEADING **is** *left*) **then** (DESIRED HEADING **is** *left*)
R5: **if** (TARGET POSITION **is** *in front*) **and** (TARGET HEADING **is** *same*) **then** (DESIRED HEADING **is** *same*)

Figure S1. The hand-tuned fuzzy knowledge base of the predator fuzzy-rule-based system.

The fuzzy-rule-based system computed the predator's desired heading direction based on its current target prey individual; it implemented classical pursuit, where the predator moves directly toward the evading target prey individual[7,8].

Let $d_t^{(i,j)} = \|\mathbf{r}_t^{(j)} - \mathbf{r}_t^{(i)}\|$ denote the distance between artificial animals i and j at time instant t and $\hat{\mathbf{d}}_t^{(i,j)} = (\mathbf{r}_t^{(j)} - \mathbf{r}_t^{(i)})/d_t^{(i,j)}$ the unit vector pointing from i to j at time instant t. The angular position of artificial animal j with respect to i is then

$$\theta_t^{(i,j)} = \arccos\left(\hat{\mathbf{v}}_t^{(i)} \cdot \hat{\mathbf{d}}_t^{(i,j)}\right) \tag{1}$$

and the relative orientation of artificial animal j with respect to i

$$\phi_t^{(i,j)} = \phi_t^{(j)} - \phi_t^{(i)}. \tag{2}$$

Let κ be a predator and α its current target. To compute the predator's desired heading its fuzzy-rule-based system input variables TARGET POSITION and TARGET HEADING were set to $\theta_t^{(\kappa,\alpha)}$ and $\phi_t^{(\kappa,\alpha)}$, respectively.

The principal focus of the study was the analysis of the evolved behaviour when varying exposure to multiple simultaneous predation pressures. We used predators that according to previous research might pressure prey towards a) grouping[9–12] (i.e. attack the most peripheral prey individual or attack the nearest prey individual) and b) dispersing[10] (i.e. attack the most central prey individual or attack high density areas).

Let $\mathrm{N}_t^{(i)} = \{j \in \mathrm{I} |\ j \neq i, d_t^{(i,j)} \leq r_\mathrm{v}^{(i)}\}$ denote the neighbourhood of prey individual i. High density area attacking predators (h) detected and pursued the prey individual with the highest amount of nearby neighbours;

$$\alpha_t^{(h)} = \arg\max_{i \in \mathrm{I}} \left|\mathrm{N}_t^{(i)}\right|. \tag{3}$$

Note that in the case of a high density area attacking predator (HDAA predator) the targeted prey individual constantly changed, so that the HDAA predator effectively detected and pursued the highest density area. Following Olson et al.[10] the HDAA predators moved at a slower speed than prey and were also less manoeuvrable. If the HDAA predator was close enough to any of the prey individuals (i.e. $d_t^{(h,i)} \leq r_\mathrm{c}^{(h)}$, $i \in \mathrm{I}$) they were marked as captured. Note also that the HDAA predator could consume any prey individual and not only the targeted prey individual, therefore mimicking predators capable of attacking and capturing several prey individuals in a single predation event[13–16].

Predators that attack the nearest, the most peripheral or the most central prey individual detect, pursue, attack and capture a single prey individual[6]. In this study we label them with the common name single-target attack or ST predators. Note that in contrast to HDAA predators, in the case of an ST predator the target prey individual was detected only on special occasions and

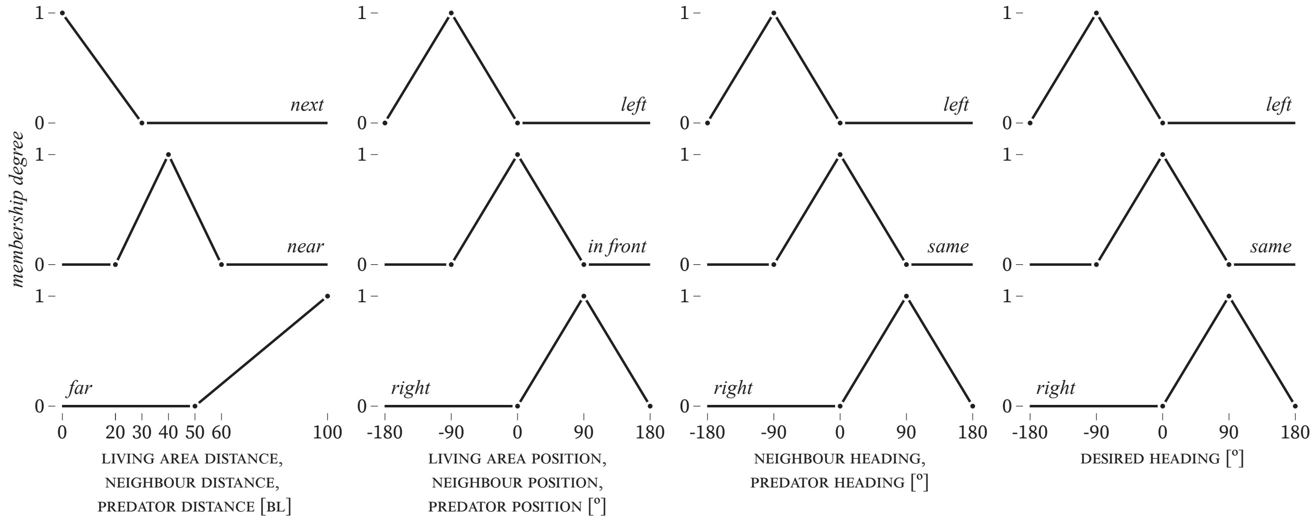

Figure S2. The data base for prey individuals. The rule base for prey individuals was generated using genetic fuzzy systems.

then pursued until captured or until the predator's hunt duration expired. The detection of the target prey individual occurred at the time instant when the ST predator appeared or the currently targeted prey individual died during pursuit.

Following previous research[6,17–19]

$$P_t^{(i)} = \frac{1}{\left|\mathrm{N}_t^{(i)}\right|} \left\| \sum_{j \in \mathrm{N}_t^{(i)}} \hat{\mathbf{d}}_t^{(i,j)} \right\| \tag{4}$$

is the peripherality of prey individual i at time instant t. The peripherality of stragglers, prey individuals with no visible neighbour, i.e. $\mathrm{N}_t^{(i)} = \emptyset$, was set to $+\infty$. Let s be an ST predator. The nearest, $\alpha_\mathrm{n}^{(s)}$, the most peripheral, $\alpha_\mathrm{p}^{(s)}$, and the most central prey individual, $\alpha_\mathrm{c}^{(s)}$, were defined as

$$\alpha_\mathrm{n}^{(s)} = \arg\min_{i \in \mathrm{I}} d_\sigma^{(s,i)}, \tag{5}$$

$$\alpha_\mathrm{c}^{(s)} = \arg\min_{i \in \mathrm{I}} P_\sigma^{(i)}, \tag{6}$$

$$\alpha_\mathrm{p}^{(s)} = \arg\max_{i \in \mathrm{I}} P_\sigma^{(i)}, \tag{7}$$

where σ is the time instant of target prey individual detection. In view of recent research by Biswas et al., which suggests that prey clumping emerges regardless of predator confusion[12], when an ST predator was close enough to its targeted prey individual (i.e. $d_t^{(s,\alpha)} \leq r_\mathrm{c}^{(s)}$, where α is the currently targeted prey) the latter was marked as captured. Meaning that in contrast to previous research[9–11,20], the possibility of the predator getting confused was not explicitly modelled. In other words, predators in our model did not change their target and remained focused throughout the whole hunt event. As a contrast to HDAA predators, ST predators were faster than prey, but were also less manoeuvrable.

Prey

Every prey individual's behaviour was governed by its own fuzzy-rule-based system, which by the application of fuzzy reasoning over an evolving set of if-then rules determined the individual's desired heading in the next time step. Only the fuzzy rule base was evolved; the fuzzy database was kept constant (see Figure S2). The input variables can be decomposed into three parts: a) information regarding the living area, b) information regarding the nearest predator, and c) information regarding the interacting neighbour.

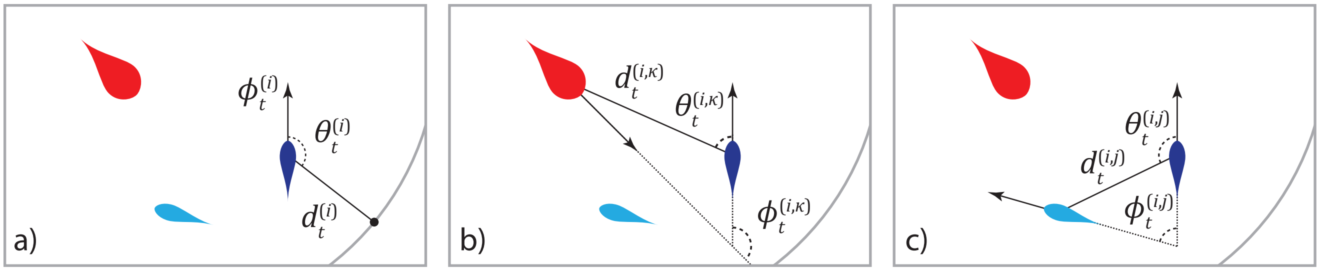

Figure S3. Quantities available to prey individuals in order to determine their desired heading: a) distance, $d_t^{(i)}$, and angular position $\theta_t^{(i)}$ of the nearest point on the border of the living area, b) distance, $d_t^{(i,\kappa)}$, angular position, $\theta_t^{(i,\kappa)}$, and relative orientation, $\phi_t^{(i,\kappa)}$, of the nearest predator, c) distance, $d_t^{(i,j)}$, angular position, $\theta_t^{(i,j)}$, and relative orientation, $\phi_t^{(i,j)}$, of the interacting neighbour.

At all times prey individuals were aware of the distance and angular position of the nearest point on the border of the living area (Figure S3a), so the input variables LIVING AREA DISTANCE and LIVING AREA POSITION of prey individual i were set to

$$d_t^{(i)} = \min(0,\ \max(r_{\mathrm{LA}} - \|\mathbf{r}_t^{(i)}\|),\ r_{\mathrm{V}}^{(i)}) \tag{8}$$

and

$$\theta_t^{(i)} = \arccos\left(\hat{\mathbf{v}}_t^{(i)} \cdot \hat{\mathbf{r}}_t^{(i)}\right), \tag{9}$$

respectively.

As empirical data suggests that animals in groups usually react to predator attacks[21,22], it is safe to assume that in most cases prey individuals can detect the incoming predator attack at some point in time. For this reason, in our model, prey individuals were aware of the nearest visible predator. From the prey individual's point of view this is the predator that poses the highest threat[23]. The input variables PREDATOR DISTANCE, PREDATOR POSITION, and PREDATOR HEADING were set to $d_t^{(i,\kappa)}$, $\theta_t^{(i,\kappa)}$, and $\phi_t^{(i,\kappa)}$, respectively (Figure S3b). Here κ denotes the nearest currently visible predator of prey individual i, computed as

$$\kappa = \arg\min_{k \in \mathrm{K}} d_t^{(i,k)},\ \mathrm{K} = \{k \in \mathrm{S} \cup \mathrm{H} |\ d_t^{(i,k)} \le r_{\mathrm{V}}^{(i)}\}, \tag{10}$$

where K is the set of predators (ST or HDAA) currently visible by prey individual i. If the prey individual did not see any predators the input variables were marked as UNDEFINED. If-then rules with any of their input variables marked as UNDEFINED were excluded from fuzzy reasoning.

A large-scale empirical study conducted in Rome[24] discovered an anisotropic nature of interactions in starling flocks. This type of interaction became known as topological interaction, where an individual interacts with ≈6.5 of its nearest neighbours. Bode et al.[25] proposed a much simpler interaction model capable of replicating the anisotropic nature of the interactions observed in the empirical study. In their model interaction is stochastic in nature and the probability of prey individual i interacting with prey individual j depends on visibility of individual j and is inversely proportional to its distance,

$$p(i,j) = \begin{cases} \frac{1}{d_t^{(i,j)}}, & \text{iff } j \in \mathrm{N}_t^{(i)} \\ 0, & \text{otherwise.} \end{cases} \tag{11}$$

Following Bode et al.[25] prey individuals in our model at every time instant interacted with only one other prey individual, as this greatly reduces the number of fuzzy-rules that have to be evaluated per prey individual. The fuzzy-rule-based system input variables NEIGHBOUR DISTANCE, NEIGHBOUR POSITION, and NEIGHBOUR HEADING were set to $d_t^{(i,j)}$, $\theta_t^{(i,j)}$, and $\phi_t^{(i,j)}$, respectively (Figure S3c). Like in the case of the input variables related to the nearest predator, if the prey individual had no visible neighbour, i.e $\mathrm{N}_t^{(i)} = \emptyset$ in eq. (11), the input variables were marked as UNDEFINED.

Evolution was achieved by the application of genetic algorithms over the fuzzy-rule-based systems, i.e. by genetic fuzzy systems. Genetic fuzzy systems have been extensively used for the optimization of hand-crafted fuzzy knowledge bases, and evolution of data bases, rule bases or the whole fuzzy knowledge base[26–28]. In our case we pre-specified the data base and used

Table S2. Parameters of the genetic fuzzy system.

Description	Default (tested) value
Number of if-then rules per individual	2–30 (2–50)
Number of antecedents per rule	1–2 (1–3)
Mutation rate	2%
Mutation intensity	3

a genetic fuzzy system to evolve only the rule bases of every prey individual. Our inspiration were messy genetic algorithms[29] which use the Pittsburgh approach: each individual in the population is a complete rule base[30,31].

The initial population of prey individuals had a randomly generated rule base. The only constraints were the number of if-then rules per individual and the number of antecedents per rule (see Table S2). The constraints were selected to keep an individual's reasoning as simple as possible. Preliminary tests of the influence of these two constraints on the resulting behaviour by performing evolutions with a higher maximal number of rules (50) and/or antecedents (3) showed results similar to those reported in the main article.

When a prey individual died a new prey individual (child) was created by merging the rule bases of two prey individuals (parents) that were still alive. Selection of parents was fitness proportionate, where the fitness was based on the prey individual's energy level. The number of rules in the rule base of the child prey individual was a uniformly distributed random number between the number of rules in the parent's rule base, whose rule base had the lowest number of rules, and the number of rules in the other parent's rule base. For example, if the rule base of one parent had 15 rules and the other 18 the child could have anywhere between 15 and 18 rules.

Rules in the rule bases of the two parents that had the same antecedent part and the same consequent part of the if-then rule were treated as equal. First equal rules were copied to the child's rule base. Following that the remaining slots were filled with random rules from the set of non-equal rules. Once the child's rule base had the required number of rules there was a 2% chance a mutation could trigger (mutation rate). There were two kinds of mutations in our genetic fuzzy system; the first type inserted new random rules into the child's rule base, the other removed existing rules. The amount of rules inserted or removed was a uniformly distributed random number between 1 and 3 (mutation intensity). Note that each mutation type could trigger independently so every time a new child prey individual was created each mutation type (insert rules, remove rules) had a 2% chance of triggering.

Biological relevance of the parameters

Where possible we attempted to set the model's parameters to biologically relevant values (see Table S3). We modelled the prey species after the Pacific Blue-eye (*Pseudomugil signifier*), a fish species that is well known for schooling[32–34]. In the case of Herbert-Read et al.[33] the body lengths (BL) of captured Pacific Blue-eyes were from 2 to 3 cm. Other studies[35] report an average BL of approximately 3.5 cm. In our case we used a body length value of 3 cm. The cruising speed of Pacific Blue-eyes is approximately 0.124 m/s $\approx$ 4 BL/s[33]. For prey visibility distance we used the equation provided by Tyrell et al.[36]. We could not find the exact spatial resolving power of the Pacific Blue-eye so we used the value of a fish of similar size, the zebrafish (*Danio rerio*)[37]. This value is relevant since fish of similar size, have similar sized retinas, and thus similar vision capabilities[38]. The zebrafish can spot an object of size 3 cm (the size of a prey individual in our model) at a distance of 330 cm. Because the zebrafish is slightly larger than the Pacific Blue-eye and fish with larger retinas can see farther[38], we set the visibility distance of prey individuals to 100 BL. As pointed out by Domenici[39], speed changes and body size play a major role in turning rates of fish. Because the speed in our model is constant we could not use the empirical data about fish turning rates. Which is why the maximum manoeuvrability of prey individuals was tuned by hand in such a way that with a hand-tuned fuzzy-rule-based system prey individuals were able to avoid others effectively but without introducing too erratic or jerky movements.

An example of a single-target attack predator that attacks schools of Pacific Blue-eyes is the flathead Gudgeon (*Philypnodon grandiceps*)[34]. The flathead Gudgeon is usually around 8 cm in length, but it can sometimes grow up to 12 cm in length[35], so we set the size of ST predators in our model to 3 BL (9 cm). The linear regression formula provided by Domenici[39] suggests that a predator that is 3 times larger than the prey is also approximately 1.4 times faster than the prey. Based on that we set the speed of ST predators in our model to 5.6 BL/s. The ST predator vision radius calculated using the same approach as for prey individual's[36–38], would be approximately 300 BL. However, in mammals[40], once the effects of eye size and phylogeny have been statistically controlled, predatory habits are associated with a higher visual acuity. In addition, some aquatic predators, e.g. swordfish (*Xiphias gladius*), warm their retina to significantly improve temporal resolution, and hence the detection of rapid motion[41]. Since the goal of this research is the investigation of behaviour that evolves under various predation pressure mixtures, we wanted to guarantee that the predators can always find and target the most vulnerable prey individual (with respect

Table S3. Default parameter values of the individual based model; ST – single-target attack, HDAA – high density area attack.

Parameter	Description	Default value
τ	Update step	1 s
BL	Body length	3 cm
r_{LA}	Living area radius	350 BL
I	Set of prey individuals (size)	100
$b^{(i)}$	Prey appearance area radius	325 BL
$s^{(i)}$	Prey size	1 BL
$v^{(i)}$	Prey speed	4 BL/s
$r_{\mathrm{v}}^{(i)}$	Prey vision radius	100 BL
$\omega_{\mathrm{max}}^{(i)}$	Prey maximum manoeuvrability	0.23 rad/s
ε_0	Prey initial energy	1000
ε_{l}	Prey living energy gain	1
ε_{c}	Prey collision penalty	-10
ε_{w}	Prey wandering penalty	-10
t_{r}	Predator re-appearance time	600–1200 s
t_{h}	Predator hunt duration	600 s
S	Set of ST predators (size)	0–8
$b^{(s)}$	ST predator appearance distance	400 BL
$s^{(s)}$	ST predator size	3 BL
$v^{(s)}$	ST predator speed	5.6 BL/s
$r_{\mathrm{c}}^{(s)}$	ST predator catch distance	3 BL
$\omega_{\mathrm{max}}^{(s)}$	ST predator maximum manoeuvrability	0.16 rad/s
H	Set of HDAA predators (size)	0–8
$b^{(h)}$	HDAA predator appearance distance	400 BL
$s^{(h)}$	HDAA predator size	12 BL
$v^{(h)}$	HDAA predator speed	3 BL/s
$r_{\mathrm{c}}^{(h)}$	HDAA predator catch distance	12 BL
$\omega_{\mathrm{max}}^{(h)}$	HDAA predator maximum manoeuvrability	0.03 rad/s
E	Duration of evolution	10.000.000 s
I	Number of iterations	20

to the predator's predation tactic). Which is why the vision of predators in our model is not limited. With this we also removed the occurrences when the predator "did not see a potential prey target". To calculate the manoeuvrability of ST predators we used the equation designed on empirical data by McKenzie et al.[42]. Using this equation, we calculated that a single target predator should be 1.7 times less manoeuvrable than prey.

References

1. Zadeh, L. Fuzzy sets. *Information and Control* **8**, 338–353 (1965).
2. Mamdani, E. Application of fuzzy algorithms for control of simple dynamic plant. *Proceedings of the Institution of Electrical Engineers* **121**, 1585–1588 (1974).
3. Mendel, J. M. *Uncertain rule-based fuzzy logic system: introduction and new directions* (Prentice–Hall, NJ, 2001).
4. Lebar Bajec, I., Zimic, N. & Mraz, M. Simulating flocks on the wing: the fuzzy approach. *J Theor Biol* **233**, 199–220 (2005).
5. Lebar Bajec, I. *Fuzzy Model for a Computer Simulation of Bird Flocking*. Ph.D. thesis, University of Ljubljana, Faculty of Computer and Information Science, Ljubljana, Slovenia (2005).
6. Demšar, J. & Lebar Bajec, I. Simulated predator attacks on flocks: a comparison of tactics. *Artif Life* **20**, 343–359 (2014).
7. Kane, S. & Zamani, M. Falcons pursue prey using visual motion cues: new perspectives from animal-borne cameras. *J Exp Biol* **217**, 225–234 (2014).

8. Nahin, P. *Chases and Escapes: The Mathematics of Pursuit and Evasion* (Princeton University Press, 2012).

9. Kunz, H., Züblin, T. & Hemelrijk, C. On prey grouping and predator confusion in artificial fish schools. In *Proceedings of the Tenth International Conference of Artificial Life*, 365–371 (MIT Press, 2006).

10. Olson, R., Hintze, A., Dyer, F., Knoester, D. & Adami, C. Predator confusion is sufficient to evolve swarming behaviour. *J R Soc Interface* **10** (2013).

11. Olson, R., Knoester, D. & Adami, C. Evolution of swarming behavior is shaped by how predators attack. *Artif Life* **22**, 299–318 (2016).

12. Biswas, R., Ofria, C., Bryson, D. & Wagner, A. Causes vs benefits in the evolution of prey grouping. In *ALIFE 14: The Fourteenth Conference on the Synthesis and Simulation of Living Systems*, vol. 14, 641–648 (2014).

13. Nøttestad, L. & Axelsen, B. Herring schooling manoeuvres in response to killer whale attacks. *Can J Zool* **77**, 1540–1546 (1999).

14. Nøttestad, L., Fernö, A., Mackinson, S., Pitcher, T. & Misund, O. How whales influence herring school dynamics in a cold-front area of the norwegian sea. *ICES J Mar Sci* **59**, 393–400 (2002).

15. Mori, M. & Butterworth, D. A first step towards modelling the krill-predator dynamics of the antarctic ecosystem. *CCAMLR Science* **13**, 217–277 (2006).

16. Goldbogen, J. *et al.* Mechanics, hydrodynamics and energetics of blue whale lunge feeding: efficiency dependence on krill density. *J Exp Biol* **214**, 131–146 (2011).

17. Hemelrijk, C. Towards the integration of social dominance and spatial structure. *Anim Behav* **59**, 1035–1048 (2000).

18. Hemelrijk, C., Wantia, J. & Gygax, L. The construction of dominance order: comparing performance of five methods using an individual-based model. *Behaviour* **142**, 1037–1058 (2005).

19. Hildenbrandt, H., Carere, C. & Hemelrijk, C. Self-organized aerial displays of thousands of starlings: a model. *Behav Ecol* **21**, 1349–1359 (2010).

20. Demšar, J., Hemelrijk, C., Hildenbrandt, H. & Lebar Bajec, I. Simulating predator attacks on schools: Evolving composite tactics. *Ecol Modell* **304**, 22–33 (2015).

21. Partridge, B. The structure and function of fish schools. *Sci Am* **246**, 114–123 (1982).

22. Pitcher, T. & Wyche, C. *Predator-avoidance behaviours of sand-eel schools: why schools seldom split*, 193–204 (Springer, 1983).

23. Rieucau, G., Boswell, K., De Robertis, A., Macaulay, G. & Handegard, N. Experimental evidence of threat-sensitive collective avoidance responses in a large wild-caught herring school. *PLoS One* **9**, 1–9 (2014).

24. Ballerini, M. *et al.* Interaction ruling animal collective behavior depends on topological rather than metric distance: Evidence from a field study. *Proc Natl Acad Sci U S A* **105**, 1232–1237 (2008).

25. Bode, N., Faria, J., Franks, D., Krause, J. & Wood, A. How perceived threat increases synchronization in collectively moving animal groups. *Proc R Soc Lond B Biol Sci* **277**, 3065–3070 (2010).

26. Cordon, O., Gomide, F., Herrera, F., Hoffmann, F. & Magdalena, L. Ten years of genetic fuzzy systems: current framework and new trends. *Fuzzy Sets Syst* **141**, 5–31 (2004).

27. Fernández, A., López, V., del Jesus, M. & Herrera, F. Revisiting evolutionary fuzzy systems: Taxonomy, applications, new trends and challenges. *Knowl Based Syst* **80**, 109–121 (2015).

28. Herrera, F. Genetic fuzzy systems: taxonomy, current research trends and prospects. *Evol Intell* **1**, 27–46 (2008).

29. Goldberg, B., D. E.and Korb & Deb, K. Messy genetic algorithms: Motivation, analysis, and first results. *Complex Systems* **3**, 493–530 (1989).

30. Smith, S. *A learning system based on genetic adaptive algorithms*. Ph.D. thesis, University of Pittsburgh, Pittsburgh, PA (1980).

31. Hoffmann, F. & Pfister, G. Evolutionary design of a fuzzy knowledge base for a mobile robot. *Int J Approx Reason* **17**, 447–469 (1997). Genetic Fuzzy Systems for Control and Robotics.

32. Herbert-Read, J., Logendran, D. & Ward, A. Sensory ecology in a changing world: salinity alters conspecific recognition in an amphidromous fish, pseudomugil signifer. *Behav Ecol Sociobiol* **64**, 1107–1115 (2010).

33. Herbert-Read, J., Buhl, J., Hu, F., Ward, A. & Sumpter, D. Initiation and spread of escape waves within animal groups. *R Soc Open Sci* **2** (2015).

34. Herbert-Read, J. The three-dimensional shape and structure of fish schools under attack from predators. In *Collective Motion 2016: Math, biology, physics and engineering come together*, 20 (Uppsala, Sweden, 2016).

35. Pusey, B., Mark, K. & A., A. *Freshwater Fishes of North-Eastern Australia* (CSIRO Publishing, Melbourne, 2004).

36. Tyrrell, L., Moore, B., Loftis, C. & Fernández-Juricic, E. Looking above the prairie: localized and upward acute vision in a native grassland bird. *Sci Rep* **3** (2013).

37. Pita, D., Moore, B., Tyrrell, L. & Fernández-Juricic, E. Vision in two cyprinid fish: implications for collective behavior. *PeerJ* **3**, e1113 (2015).

38. Hairston, N., Li, K. & Easter, S. Fish vision and the detection of planktonic prey. *Science* **218**, 1240–1242 (1982).

39. Domenici, P. The scaling of locomotor performance in predator–prey encounters: from fish to killer whales. *Comp Biochem Physiol A Mol Integr Physiol* **131**, 169–182 (2001).

40. Veilleux, C. & Kirk, E. Visual acuity in mammals: effects of eye size and ecology. *Brain Behav Evol* **83**, 43–53 (2014).

41. Fritsches, K., Brill, R. & Warrant, E. Warm eyes provide superior vision in swordfishes. *Curr Biol* **15**, 55–58 (2005).

42. D.J., M., M.E., H. & P., D. Locomotion in primitive fishes. In *Primitive Fishes*, vol. 26 of *Fish Physiology*, 319–380 (Academic Press, 2007).

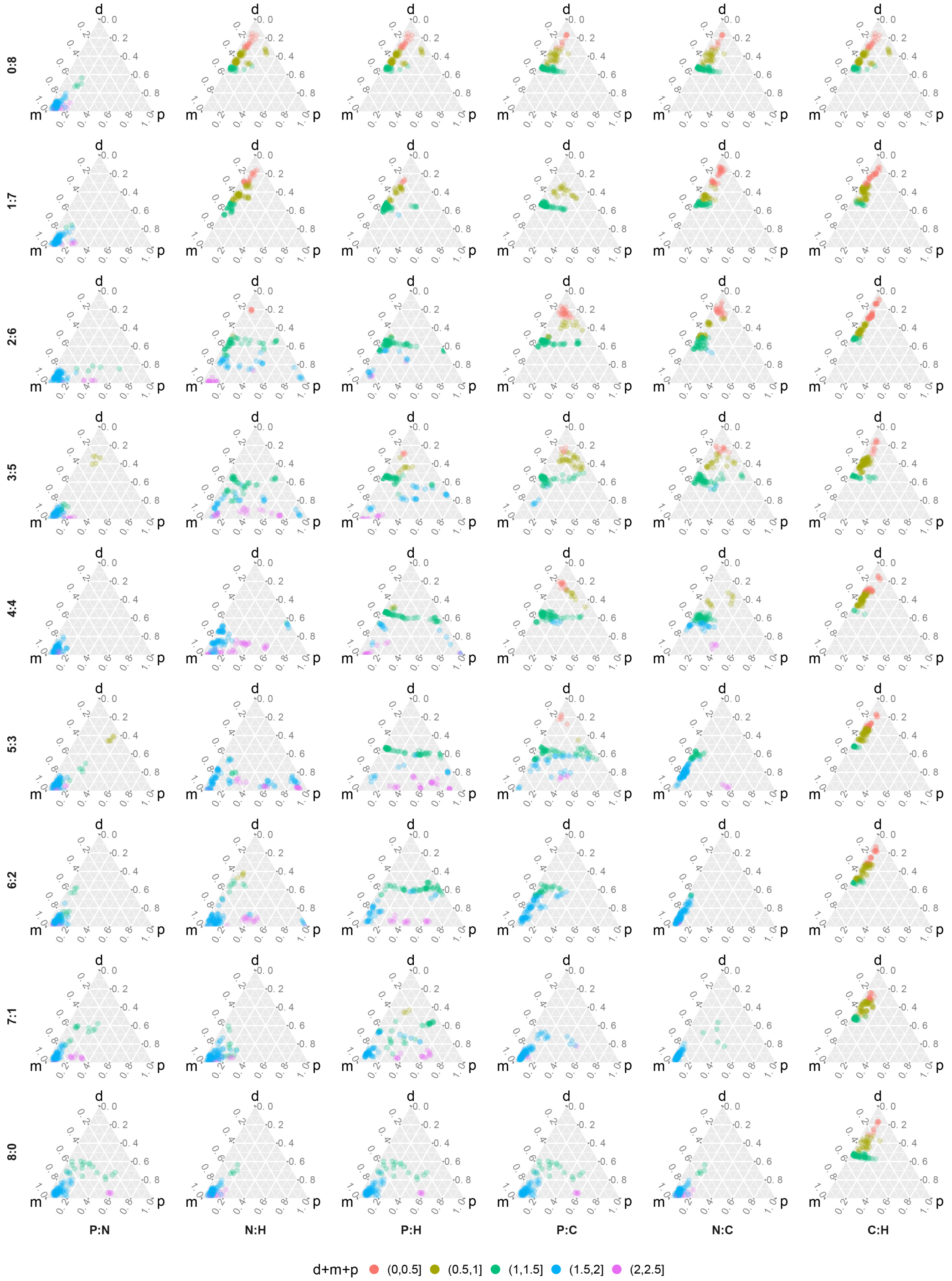

Figure S4. Normalized prey density (d), polarization (p) and angular momentum (m) for all predation pressure mixtures, N=5400; P – attack prey individuals located at the periphery of the prey groups, N – attack the nearest prey individual, C – attack the most central prey individual in a prey group, and H – high density area attacks. The predator pressure combinations and mixtures were ordered so that the top row and left column, represent the highest pressure towards grouping, the right column and bottom row the highest pressure towards dispersing. The middle section represents antagonistic predation pressures. The scales of the ternary plot were arranged so that the top corner indicates low density, the bottom left corner indicates high density and angular momentum, and the bottom right corner indicates high density and polarization. Note that the ternary plot shows the relationship between the three variables (which one dominates, in relative terms). Since the same point can represent completely different behaviours the sum of the three dimensions is used for colour coding.

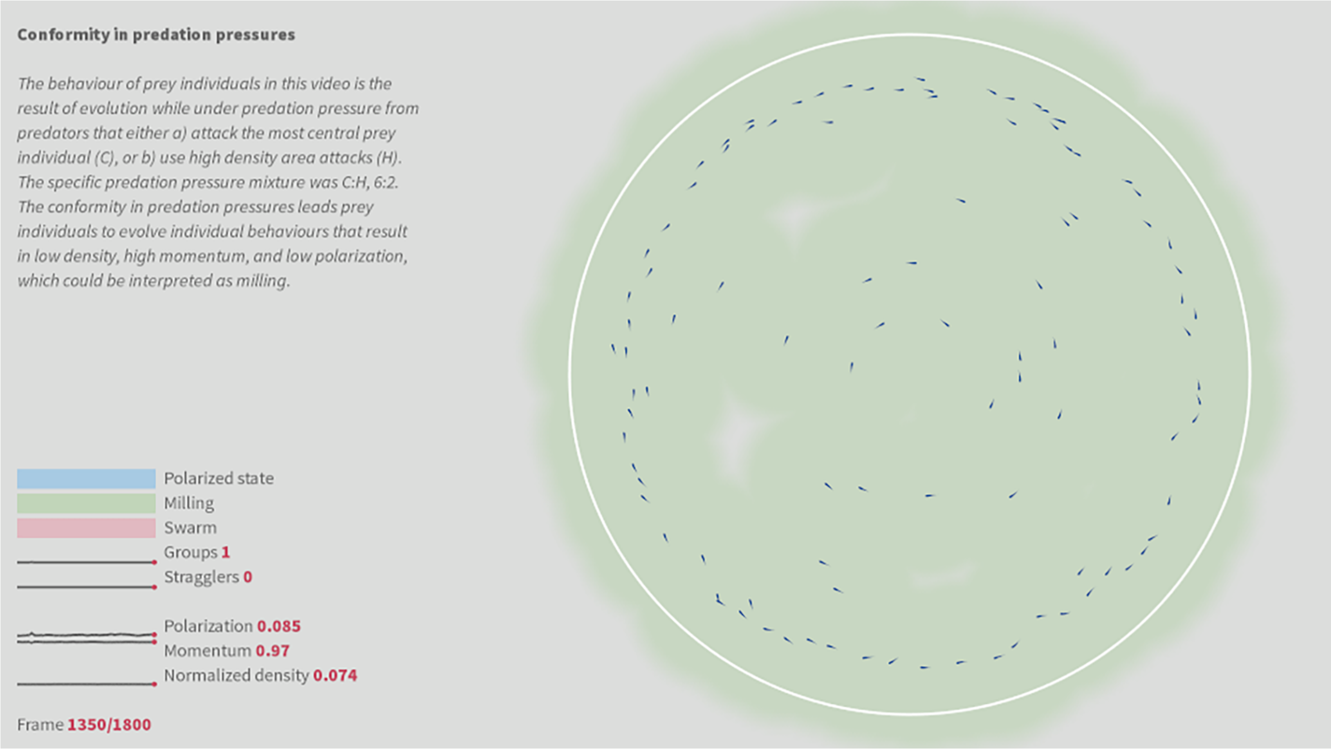

Video S1. Conformity in predation pressures. The behaviour of prey individuals in this video is the result of evolution while under predation pressure from predators that either a) attack the most central prey individual (C), or b) use high density area attacks (H). The specific predation pressure mixture was C:H, 6:2. The conformity in predation pressures leads prey individuals to evolve individual behaviours that result in low density, high momentum, and low polarization, which could be interpreted as milling.

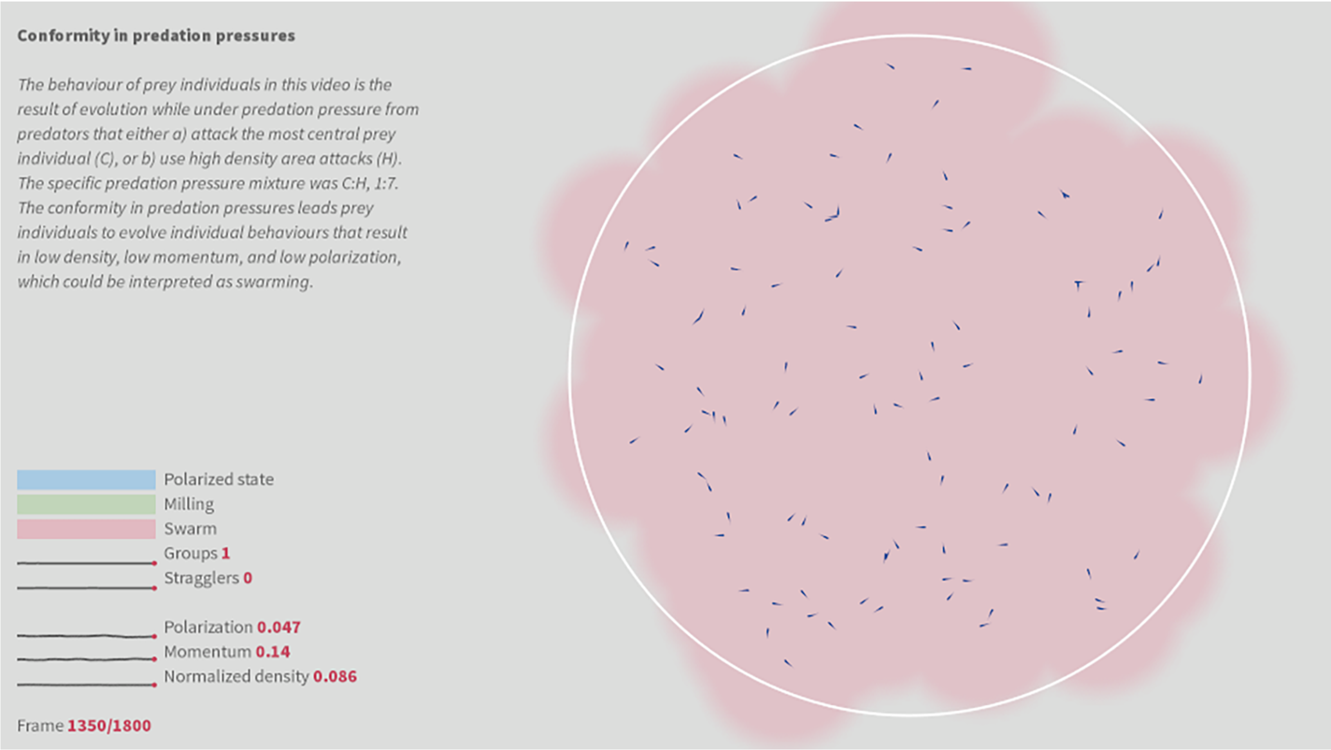

Video S2. Conformity in predation pressures. The behaviour of prey individuals in this video is the result of evolution while under predation pressure from predators that either a) attack the most central prey individual (C), or b) use high density area attacks (H). The specific predation pressure mixture was C:H, 1:7. The conformity in predation pressures leads prey individuals to evolve individual behaviours that result in low density, low momentum, and low polarization, which could be interpreted as swarming.

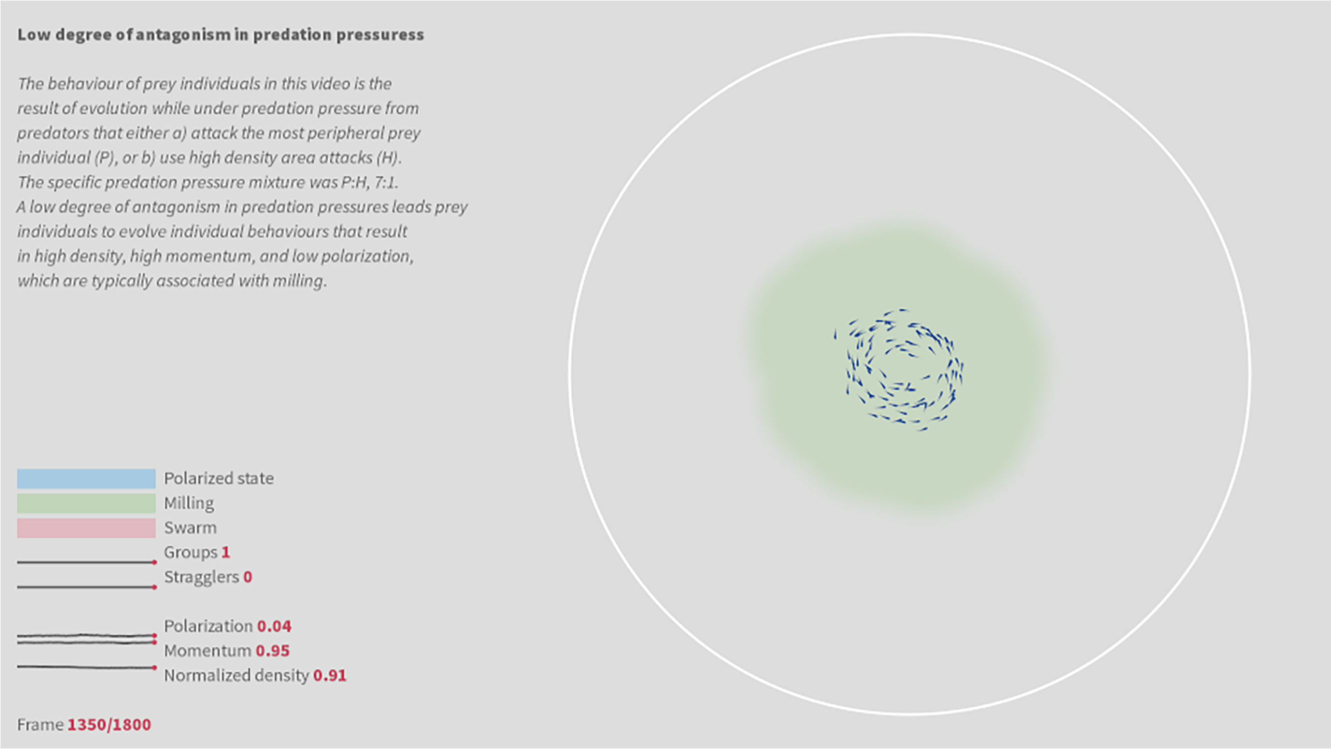

Video S3. Low degree of antagonism in predation pressuress. The behaviour of prey individuals in this video is the result of evolution while under predation pressure from predators that either a) attack the most peripheral prey individual (P), or b) use high density area attacks (H). The specific predation pressure mixture was P:H, 7:1. A low degree of antagonism in predation pressures leads prey individuals to evolve individual behaviours that result in high density, high momentum, and low polarization, which are typically associated with milling.

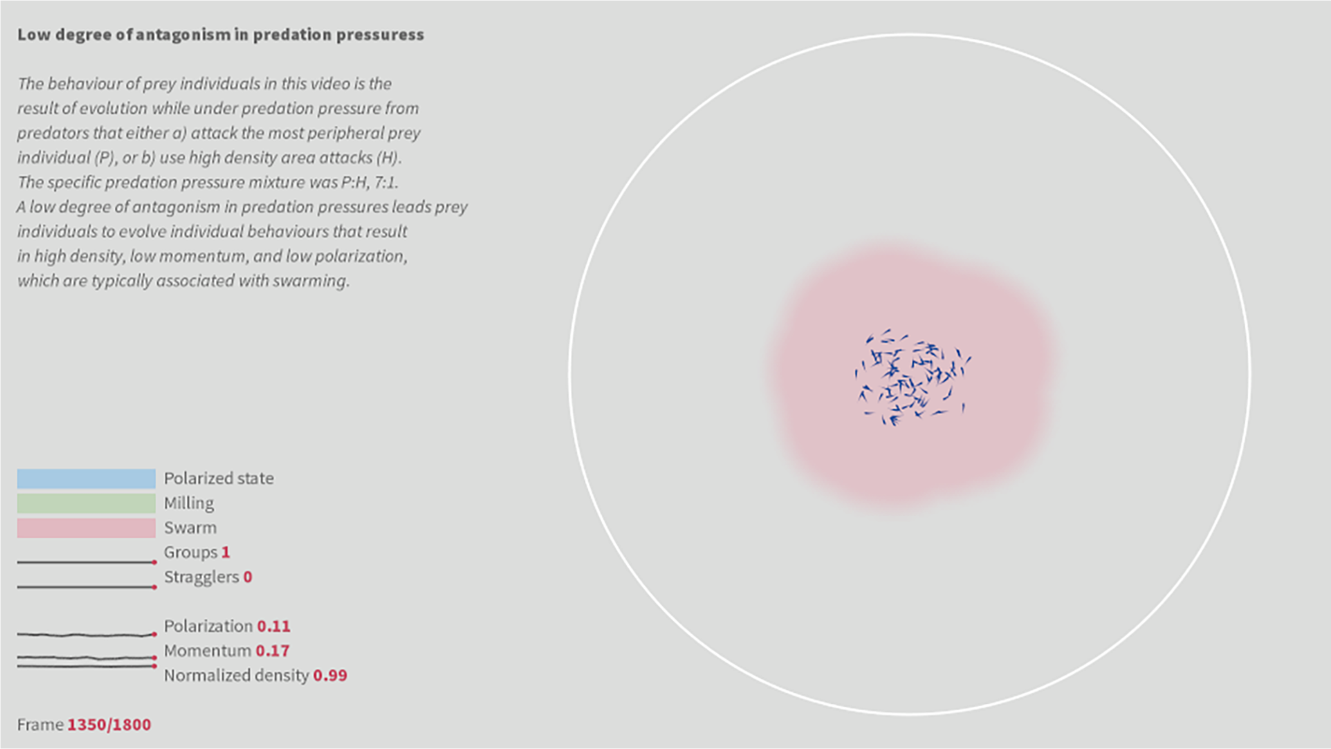

Video S4. Low degree of antagonism in predation pressuress. The behaviour of prey individuals in this video is the result of evolution while under predation pressure from predators that either a) attack the most peripheral prey individual (P), or b) use high density area attacks (H). The specific predation pressure mixture was P:H, 7:1. A low degree of antagonism in predation pressures leads prey individuals to evolve individual behaviours that result in high density, low momentum, and low polarization, which are typically associated with swarming.

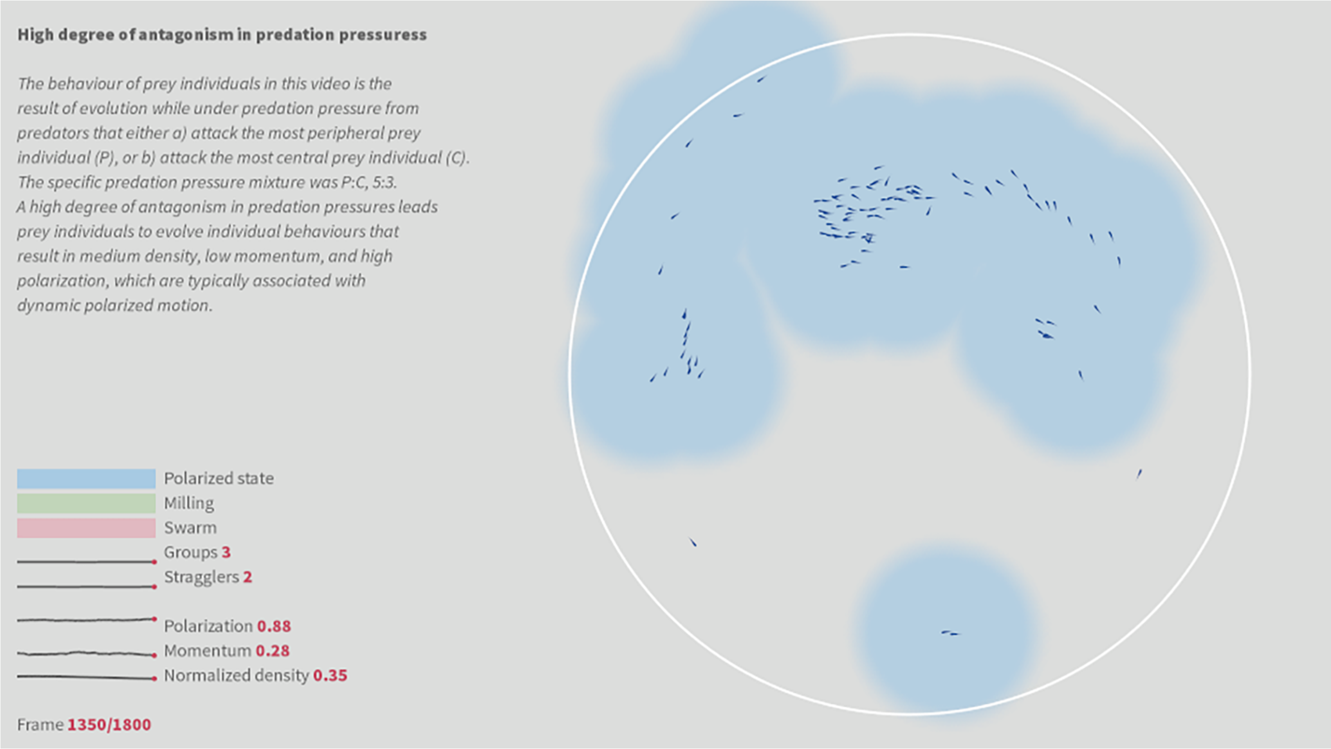

Video S5. High degree of antagonism in predation pressuress. The behaviour of prey individuals in this video is the result of evolution while under predation pressure from predators that either a) attack the most peripheral prey individual (P), or b) attack the most central prey individual (C). The specific predation pressure mixture was P:C, 5:3. A high degree of antagonism in predation pressures leads prey individuals to evolve individual behaviours that result in medium density, low momentum, and high polarization, which are typically associated with dynamic polarized motion.

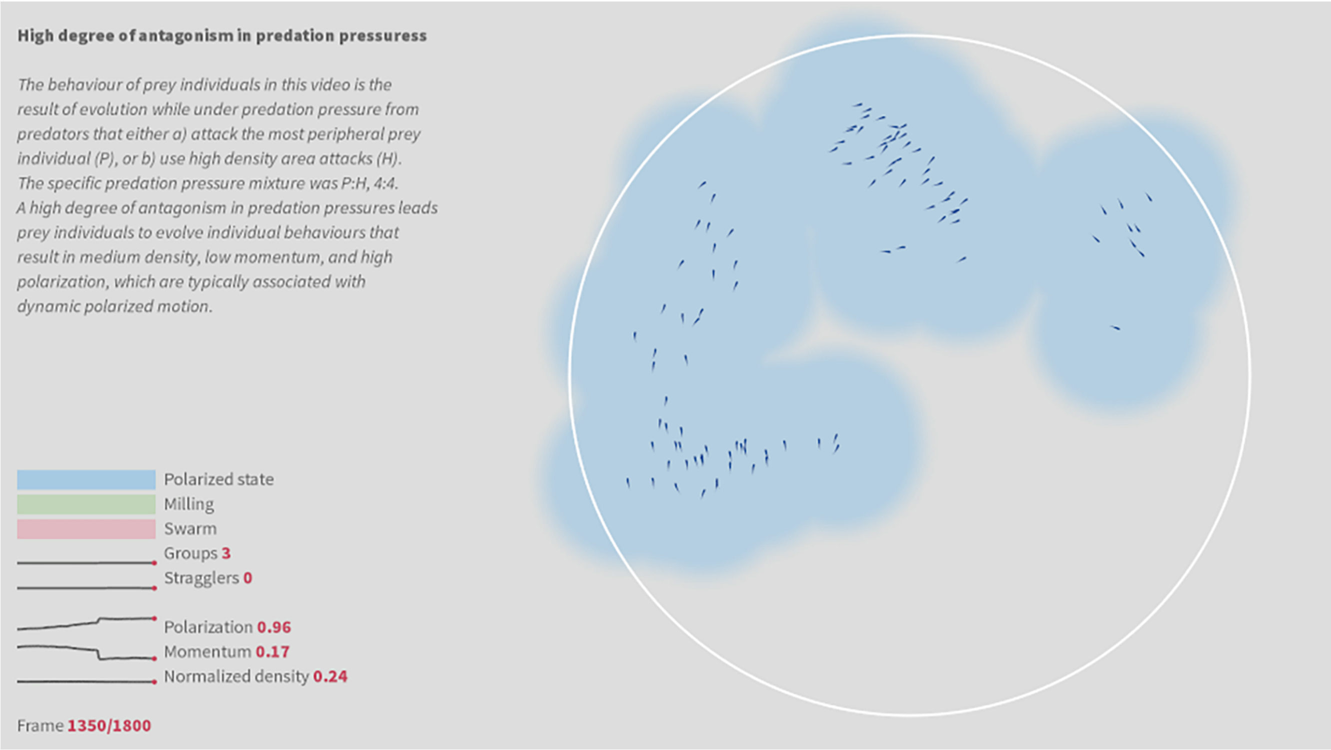

Video S6. High degree of antagonism in predation pressuress. The behaviour of prey individuals in this video is the result of evolution while under predation pressure from predators that either a) attack the most peripheral prey individual (P), or b) use high density area attacks (H). The specific predation pressure mixture was P:H, 4:4. A high degree of antagonism in predation pressures leads prey individuals to evolve individual behaviours that result in medium density, low momentum, and high polarization, which are typically associated with dynamic polarized motion.

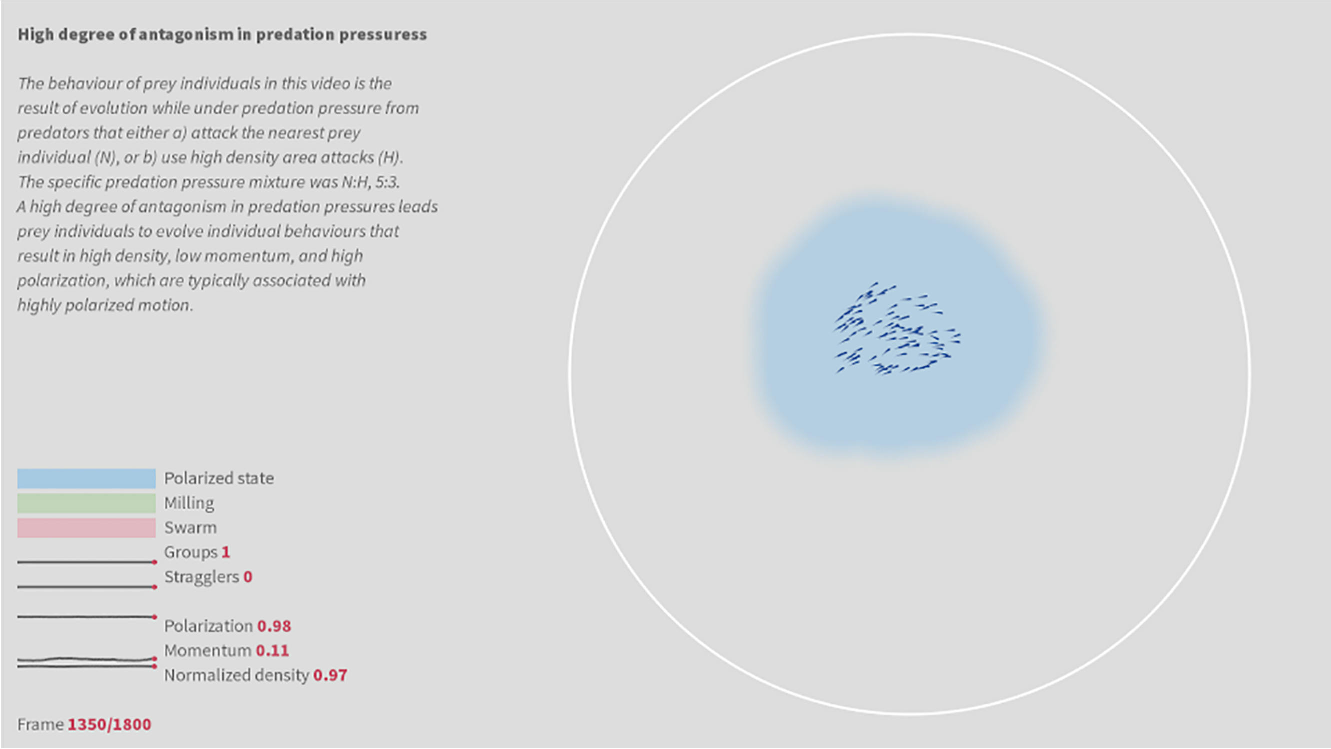

Video S7. High degree of antagonism in predation pressuress. The behaviour of prey individuals in this video is the result of evolution while under predation pressure from predators that either a) attack the nearest prey individual (N), or b) use high density area attacks (H). The specific predation pressure mixture was N:H, 5:3. A high degree of antagonism in predation pressures leads prey individuals to evolve individual behaviours that result in high density, low momentum, and high polarization, which are typically associated with highly polarized motion.
